# How Far Does a Receptor Influence Vibrational Properties of an Odorant?

**DOI:** 10.1371/journal.pone.0152345

**Published:** 2016-03-25

**Authors:** Anna Reese, Nanna Holmgaard List, Jacob Kongsted, Ilia A. Solov’yov

**Affiliations:** Department of Physics, Chemistry and Pharmacy, University of Southern Denmark, Campusvej 55, DK-5230 Odense M, Denmark; Alexander Fleming Biomedical Sciences Research Center, GREECE

## Abstract

The biophysical mechanism of the sense of smell, or olfaction, is still highly debated. The mainstream explanation argues for a shape-based recognition of odorant molecules by olfactory receptors, while recent investigations suggest the primary olfactory event to be triggered by a vibrationally-assisted electron transfer reaction. We consider this controversy by studying the influence of a receptor on the vibrational properties of an odorant in atomistic details as the coupling between electronic degrees of freedom of the receptor and the vibrations of the odorant is the key parameter of the vibrationally-assisted electron transfer. Through molecular dynamics simulations we elucidate the binding specificity of a receptor towards acetophenone odorant. The vibrational properties of acetophenone inside the receptor are then studied by the polarizable embedding density functional theory approach, allowing to quantify protein-odorant interactions. Finally, we judge whether the effects of the protein provide any indications towards the existing theories of olfaction.

## Introduction

Through the five basic senses—hearing, taste, vision, touch and smell—animals and human beings are able to perceive their environment. The molecular mechanisms of the first four of these senses are believed to be well understood and their descriptions can be found in many biochemistry textbooks [1, 2]. However, the fundamental mechanism of smell, or olfaction, is still somewhat unclear. Moreover, it is highly debated how different olfactory receptors (ORs) detect odorants [3–9] and two different explanations have evolved at present. One argues for a so-called lock-and-key mechanism [9–12], where the olfactory receptor is forced to change its conformation upon odorant binding thereby leading to signalling. The other postulates a vibrationally-assisted odour perception [13–15]. The lock-and-key mechanism relies on the recognition of size and shape of an odorant as the sole factors to distinguish between different odorant molecules. However, in 2011 Franco *et al.*[6] demonstrated that fruit flies (*Drosophila melanogaster*) can differentiate between regular odorants and their deuterated isotopologues. Two years later Gane *et al.*[7] published that also humans are capable of discerning musk molecules from their deuterated variants. As deuteration does not change the shape of a molecule the lock-and-key criterion is insufficient to explain those findings. However, since the vibrational properties of odorants and their deuterated isotopologues are different, it was suggested [6, 7, 15] that both shape and vibrations of the odorant could contribute to odour perception.

Early theories for explaining the mechanism of olfaction were proposed some decades ago, arguing for a spectral character of the sense similar to vision and hearing [13, 14]. Inspired by this work Turin suggested a mechanism based on inelastic electron tunnelling, which occurs between a donor (D) and an acceptor (A) site of a putative olfactory receptor [15]. According to this suggestion, the energy difference between the D and A sites of the receptor, Δ*ϵ*, is too large for the electron to tunnel in the absence of an odorant. Upon binding of an odorant, inelastic tunnelling could occur through emission of an odorant phonon. The physical viability of this mechanism was tested in a series of follow-up studies [4, 5, 8, 16, 17]. All of these studies consider a simple physical model where the properties of the odorant and the receptor are not influenced by each other, i.e., neglecting the implications of the receptor on the odorant. The latter fact raised a critique by Block *et al.*[18]: they could not provide any experimental evidence for the vibrationally-assisted electron transfer in ORs and pointed out three critical aspects of the theory, neglected in earlier considerations: (i) the mechanism by which electrons are delivered to the receptor is not reliable; (ii) electron transfer mechanisms are sensitive to bonding and average fields from the environment; (iii) the assumptions about the fluctuations in the environment are not realistic. It is unfortunately hardly possible to address all these critical points at present as no atomistic structure of an OR is presently available. The critical paper by Block *et al.*[18] has triggered an ongoing discussion in the field [19, 20], and has, therefore, stipulated the present investigation.

The aim of the present study is to fill in some of the missing gaps of earlier investigations and extend the model introduced previously [4, 16] where the odorant-receptor association was modelled as an isolated odorant in a uniform electric field set up by the electron residing at either the donor or acceptor site of the receptor. In the present investigation we go beyond this simplified model and consider the odorant bound inside a real protein structure. This allows us to quantify the influence of the receptor on the electron-phonon coupling, being one of the factors involved in the suggested vibrationally-assisted mechanism of olfaction. The present paper, thus, addresses some of the critical questions pointed out by Block *et al.*; namely, point (ii), which is the only question that at present can be addressed accurately, without knowing the precise structure of the receptor. Furthermore, point (iii) will be discussed in qualitative terms.

ORs belong to the family of rhodopsin-like G-protein-coupled receptors (GPCRs) [21], but with no crystal structures of ORs presently available, the study of the biophysical mechanism of olfaction is rather involved. Several homology models for ORs have been presented [12, 22–28], obtained through aligning known amino acid sequences of ORs to other rhodopsin-like GPCRs with known crystal structures. Those homology models have been widely applied for studies of OR activation according to the lock-and-key mechanism by molecular dynamics (MD) simulations over picoseconds [23], up to several tens[24, 25] and hundreds[26] of nanoseconds time scales. A related study was recently accomplished by Park *et al.*[29] who have crystallized and characterized the active conformation of the retinal-free rhodopsin apoprotein. It was found that it exhibits properties, which are also expected for ORs [29], like the capability of binding hydrophobic ligands and forming defined hydrogen bond patterns between odorant and receptor [25, 29]. Hence, the rhodopsin-based model seems to be a promising structural model for an olfactory receptor and makes it possible for us now to study at the atomistic level how far the vibrational properties of odorants are affected by the OR.

**Fig 1:**
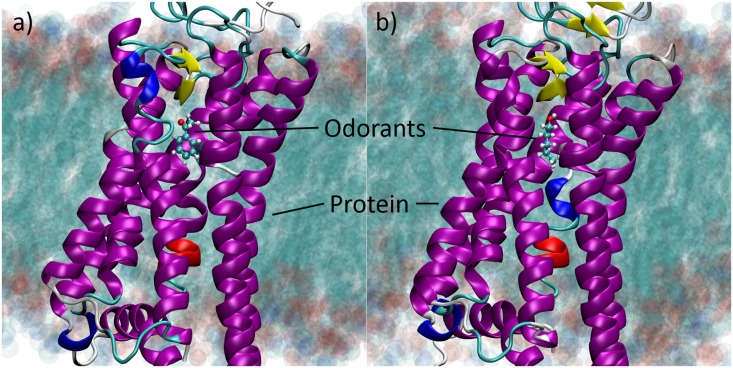
The model system. The retinal-free rhodopsin apoprotein used to model a generic OR with the bound odorants a) acetophenone and b) pentanol embedded in a lipid membrane.

In the present study we use rhodopsin-based apoprotein as model for an OR and find in a **first step** a suitable odorant which binds to the receptor in a stable fashion. Acetophenone and pentanol were tested as odorants. Fig 1 shows their location inside the OR model embedded in a lipid membrane. The odorants were tested against stable binding through extensive MD simulations exceeding 150 ns, being substantially longer than many of those previously published for different homology models of ORs [23–25]. In a **second step**, we have investigated the vibrational properties of acetophenone inside the receptor. In particular we have systematically studied how the vibrational properties of the embedded odorant are influenced by the protein scaffold. To probe the feasibility of an electron transfer (ET) reaction in the receptor we have further calculated the electron-phonon couplings, or in other words the Huang-Rhys factors [30], for each vibrational mode of the odorant and compared the results to previous estimations [4, 16]. Finally, in a **third step**, we have considered post ET events, such as OR reorganisation, and studied whether the binding mode of acetophenone inside the receptor remains stable once the electrostatics in the binding cavity is perturbed.

The focus of this study is not to prove of disprove the vibrationally-assisted olfaction mechanism, but rather to investigate to what extent a receptor could possibly influence the vibrational properties of an odorant upon a hypothesized ET event. This knowledge is currently missing but it is crucially important for supporting the vibrationally-assisted mechanism of olfaction, or proving it to be irrelevant for realistic biological systems.

## Methods

In this section we describe the details of the computational methods employed and provide information about the procedure of the simulations. Extensive classical all-atom MD simulations were performed in order to study the dynamical behaviour of the odorants and the receptor. For the calculations of the vibrational properties of acetophenone in the receptor the polarizable embedding (PE) density functional theory (DFT) method [31–33], a quantum mechanics/molecular mechanics (QM/MM) approach, was applied.

### System preparation

In the present investigation we have used the X-ray structure of the active conformation of the rhodopsin apoprotein, opsin (PDB entry: 4J4Q [29]). This structure was chosen in particular, as it turned out to demonstrate binding of a specific odorant (acetophenone) on time scales exceeding 100 ns. With MD simulations we aim to demonstrate a certain binding specificity of the model OR, which can effectively be achieved through a single MD simulation for each of the two different odorants. Two alternative homology models were also studied in a pilot investigation [27, 28], however, no binding specificity could be established on time scales less than 10 ns. Several of the probed odorants either did not bind inside the receptor models, or the receptor got filled with water; a behaviour hardly possible for a realistic OR. We, therefore, limit our present investigation to the recently suggested model of the rhodopsin apoprotein [29].

The protein preparation was performed using the Protein Preparation Wizard [34] in the graphical interface Maestro [35] implemented in the Schrödinger Suite. Hydrogen atoms were added to the structure in accordance with physiological pH. Hence, all Lys and Arg residues were positively charged while all Asp and Glu were negatively charged. Protonation states and tautomeric forms of the His imidazole rings were determined using the Protein Assignment and confirmed by visual inspection. Finally, the coordinates of the hydrogen atoms were optimized using the OPLS-2005 force field [36] while freezing the positions of the heavy atoms. Acetophenone and pentanol molecules were docked into the all-atom model of the receptor using Glide [37].

The constructed all-atom model of the receptor with either acetophenone or pentanol was embedded in a phosphatidylcholine (PC 16:0/18:1) lipid bilayer membrane patch, consisting of 154 lipids. The protein and lipids were solvated within a water box at a salt (NaCl) concentration of 0.05 mol/L and neutralizing the entire system with the salt ions. The resulting system (79.6 × 73.7 × 112.0 Å^3^) consisted of ∼68,000 atoms, including receptor with odorant molecules, lipids, water molecules, and ions. The lipid patch used here was adopted from a previous MD study [38], where as part of system preparation protocol, a protein-free membrane was simulated over 360 ns, to achieve a constant area per lipid value, which indicates a preequilibrated state of the membrane.

### MD simulations prior ET

MD simulations for (i) acetophenone and (ii) pentanol inside the receptor model were performed using NAMD 2.9 [39] with the CHARMM36 force field for proteins with CMAP corrections [40, 41], lipids [42] and employing the TIP3P water model [43]. CGenFF force-field parameters for acetophenone and pentanol were assumed [44].

Periodic boundary conditions were adopted in all MD simulations and the particle-mesh Ewald (PME) summation method was employed for evaluating Coulomb forces. The van der Waals (vdW) energy was calculated using a smooth cutoff of 12 Å. The integration time step was 2 fs; the temperature was kept at 310 K by applying Langevin forces with a damping coefficient of 5.0 ps^−1^ to all atoms in the system, except hydrogens. Each simulated system was first energy-minimized, then heated to 310 K. After heating, each system was equilibrated for 1 ns with harmonic restraints applied to the protein under NPT ensemble conditions and using Nosé-Andersen Langevin piston pressure control [45], allowing the systems to acquire a constant volume at 1 atm pressure. Next, the system was equilibrated for additional 5 ns with harmonic restraints applied only to the backbone atoms of the protein, allowing the side chains of the protein to adopt more favourable conformations, while preserving the secondary structure of the receptor model. With restraints turned off, each system was then subjected to a final 10 ns equilibration under NPT ensemble conditions. This equilibration procedure was sufficient to ensure embedding of the OR inside the pre-equilibrated membrane. Following equilibration, a 150 ns MD simulation was carried out in the NVT ensemble for the two studied systems.

### QM/MM calculations of vibrations inside the protein

To study the effect of the protein scaffold on the vibrational properties of acetophenone, and on the putative ET chain inside the receptor, the molecular system was partitioned into a quantum mechanical (QM) region consisting of acetophenone and a classical region containing the full protein and the nearest water molecules, lipids and ions (within 5 Å of the protein). The embedding potential representing the classical region consists of AMBER [46] Lennard-Jones 12–6 parameters as well as distributed permanent electric multipole moments up to and including quadrupoles and distributed anisotropic electronic dipole—dipole polarizabilities. The two latter sets of parameters were derived using the LoProp approach [47, 48] implemented in the MOLCAS version 7.8 [49, 50] program along with the molecular fractionation with conjugated caps (MFCC) fragmentation scheme, [48, 51] employing the B3LYP [52–54] exchange-correlation functional and the aug-cc-pVDZ [55] basis set. This basis set was recontracted to an atomic natural orbital type basis as required for the LoProp approach [47]. The generation of the embedding potentials was facilitated by the PE Assistant Script (PEAS) [56].

Geometry optimizations, required for the vibrational property calculations were performed on 10 statistically independent MD snapshots using the PE–DFT [31] model, as implemented in a local version of DALTON2013 [57, 58]. Hereby, acetophenone was geometry optimized in the embedding potential of the classical region, while freezing the positions of the protein and waters at the configuration at 310 K obtained from the MD simulation. Subsequently, harmonic vibrational frequencies of protein-bound acetophenone were computed by diagonalization of the mass weighted molecular Hessian for the QM region. LJ parameters for the QM region were taken from an earlier investigation [59]. All vibrational properties were computed at the B3LYP/cc-pVDZ [55] level of theory.

### Modelling of ET state

In order to mimic the structural changes of the receptor that are expected to arise upon an ET event coupled to acetophenone vibrations, we have selected a pair of amino acids located favourably with respect to acetophenone binding to participate in the ET; these amino acids were then assigned to the roles of electron donor and acceptor in the receptor. In the present model, we consider Y268 as electron donor and M86 as electron acceptor. To model the state of the receptor immediately after the ET event one electron was transferred from Y268 to M86, forming a radical pair. Hence, Y268 was modelled as a radical cation, chosen because of its modest ionisation energy, while M86 formed a radical anion and was chosen as it is situated close to acetophenone and opposes Y268 in the binding site. Even though methionine does not possess a most favourable ionisation potential to serve as an electron acceptor, the studied OR model lacks any better candidate close to the odorant binding site. The tyrosine/methionine donor/acceptor pair is also somewhat consistent with an earlier investigation on ET in methionine enkephalin, where radiolysis experiments suggested that an ultrafast ET takes place from the C-terminal tyrosine residue to the N-terminal, oxidized, methionine residue [60]. Note, that the specific choice of the donor and acceptor molecules is primarily driven by their proximity to the bound odorant, which we believe is the decisive factor if realistic ORs operate upon ET events.

In an alternative model of the ET state we consider the acceptor residue as hC86^−^ (deprotonated homocysteine), which emerged from M86 upon dissociation of a ${\text{CH}}_{3}^{•}$ radical upon ET from Y268. Such a reaction happens instantaneously for methionine in isolation, and, therefore, at certain conditions could also take place inside the protein. We, thus, for the sake of completeness, have considered hC86^−^ as a second possible electron acceptor state of the receptor.

The vibrational properties were then calculated in the same manner as described in the previous section, however, performed only for a single MD snapshot.

### MD simulations post ET

MD simulations for acetophenone inside the receptor were performed for a modified configuration of the receptor that represents a possible post ET state. For that purpose, the residues Y268 and M86 were substituted by Y268^•+^ and hC86^−^ that represent generic electron donor/acceptor partners. The force field parameters obtained using the fftk plugin [61] in VMD [62] for the charged residues are provided in the Supporting Information.

The protocol used to simulate the post ET state of the odorant-receptor system was identical to the one employed in the simulations of the prior ET state.

## Results

In this section we present and discuss the results of the MD simulations of acetophenone and pentanol inside the model OR and demonstrate long time binding for acetophenone by analysing the time dependencies of bond lengths, root-mean-square displacement (RMSD) and binding energies between the odorants and the rest of the system. Subsequently, we discuss the findings on the vibrational properties of acetophenone inside the receptor in respect of the vibrational spectrum and the Huang-Rhys factors. Finally we argue about the impact of the putative ET on odorant binding stability.

### Dynamical behaviour of the odorants in the receptor prior ET

**Fig 2:**
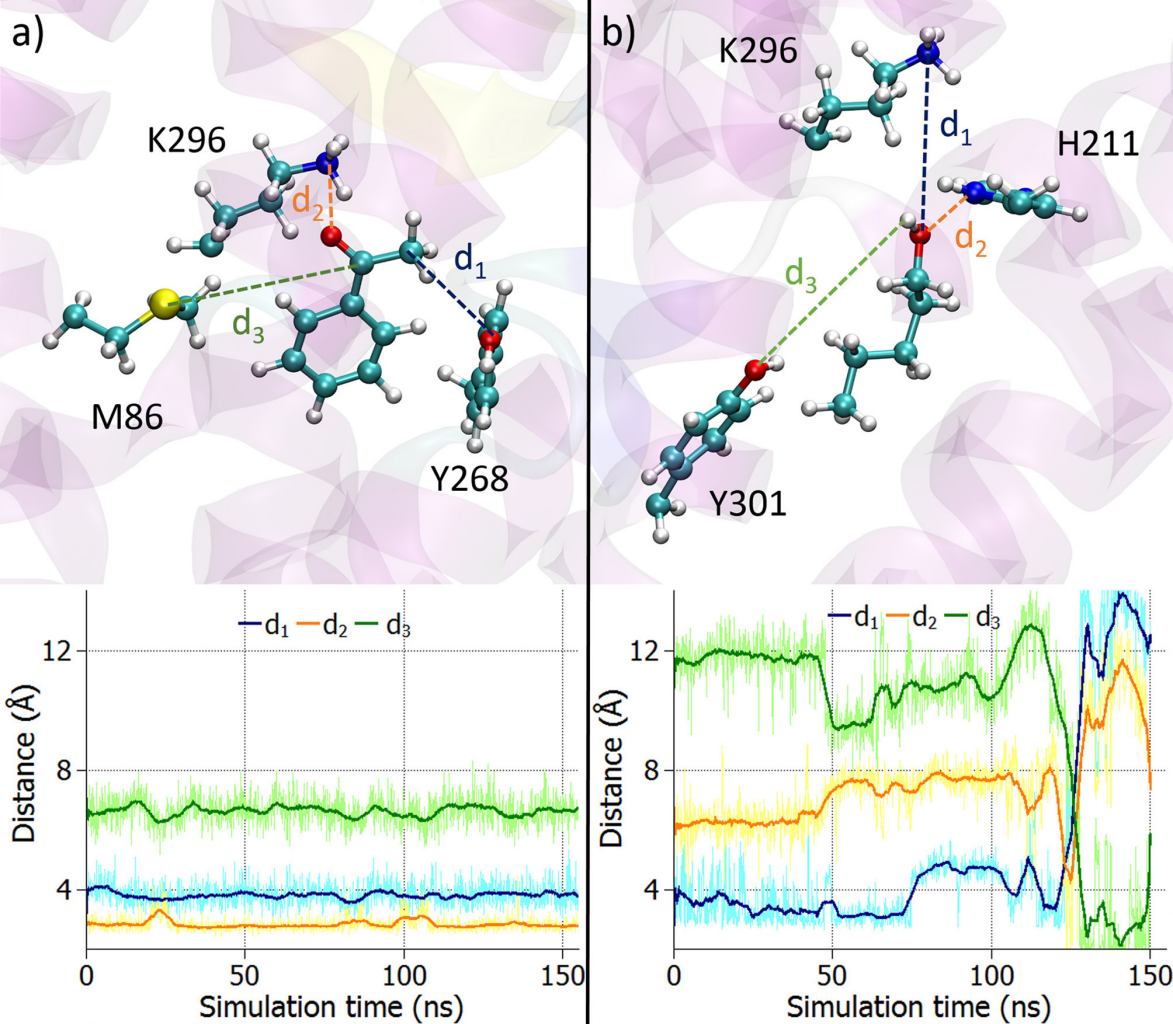
Acetophenone and pentanol binding inside the receptor. a) Acetophenone binding inside the receptor together with the side chains of the surrounding amino acids K296, Y268 and M86. The time evolution of the labelled distances *d*_1_, *d*_2_ and *d*_3_ is presented in the lower panel. b) Pentanol inside the same receptor with the side chains of the surrounding amino acids K296, Y301 and H211. The time evolution of the labelled distances *d*_1_, *d*_2_ and *d*_3_ is shown in the lower panel. The pale coloured graphs show the original data whereas the darker lines represent averaged data over 50 time steps.

We have first analysed the dynamical behaviour of the odorants inside the receptor. For that purpose, three amino acids surrounding the binding site were chosen for each odorant and the distances between those were calculated over the entire simulation. Fig 2 shows the two odorants and the side chains of these amino acid residues. The corresponding time dependencies of the labelled distances are plotted in the lower part of the figure. For acetophenone the main hydrogen bond is the one between the (protonated) amino group of residue K296 and the carbonyl oxygen of acetophenone, see distance *d*_2_ in Fig 2a. As the amino acids Y268 and M86 were assigned the roles of electron donor and acceptor, respectively (see below), they are also shown in Fig 2a. While the three distances *d*_1_, *d*_2_ and *d*_3_, characterizing acetophenone binding, remain stable over the entire simulation time, no real hydrogen bonds can be identified for pentanol in the course of the 150 ns long simulations. In fact, the measured distances between pentanol and the three amino acids initially close to the odorant fluctuate significantly with variations up to 8 Å (see Fig 2b).

**Fig 3:**
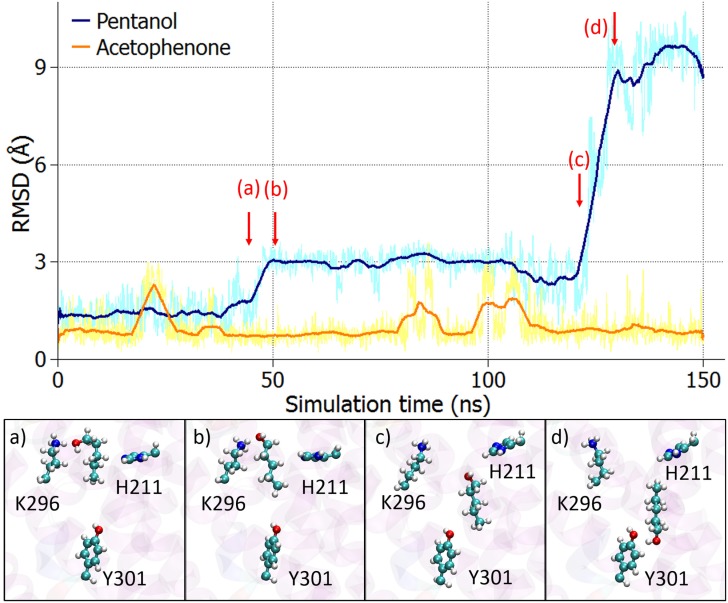
Pentanol does not show stable binding in the receptor. Time evolution of the RMSD calculated for the two odorants (acetophenone and pentanol) in respect to the post equilibration position of the odorants (see methods). While acetophenone remains stable within the receptor, pentanol shows a more flexible behaviour. The snapshots in the lower panel show the behaviour of pentanol within the receptor at the marked time instances. The original data is shown in pale colours whereas the darker lines represent averaged data over 50 time steps.

Poor binding of pentanol and reasonably persistent binding of acetophenone is supported by the RMSD analysis. The protein backbone was structurally aligned for each MD simulation and afterwards the RMSD was calculated for each odorant as a function of time relative to the post equilibration geometry of the system, see Fig 3. While the RMSD for acetophenone does not exceed a value of 3 Å, the RMSD for pentanol grows significantly with time. The increase at 45 ns is caused by the change in conformation of pentanol where it switches from a gauche-like conformation, see Fig 3a, to an all-trans conformation, depicted in Fig 3b. The flip of the molecule, illustrated in Fig 3c and 3d, within the receptor is responsible for the further increase of the RMSD at around 125 ns.

**Fig 4:**
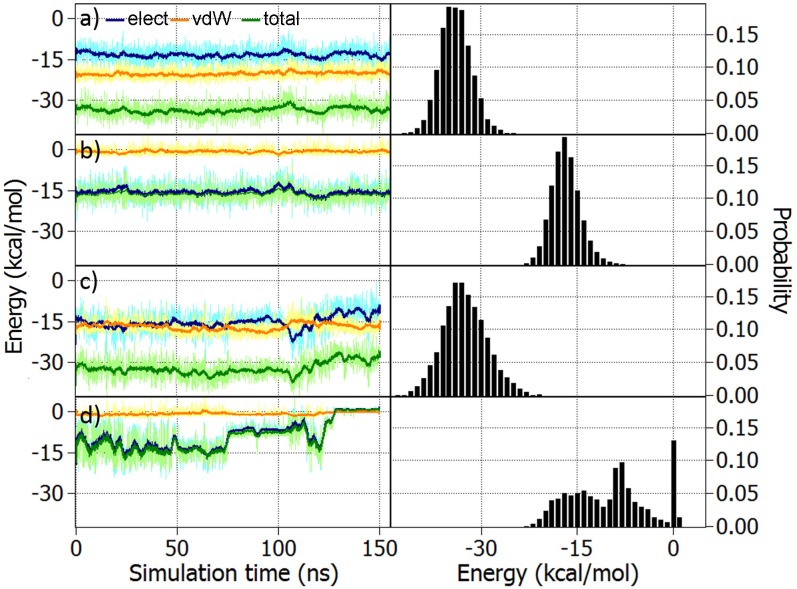
Acetophenone and pentanol binding energies. a) Electrostatic (elect, blue), van der Waals (vdW, orange) and the total non-covalent (total, green) interaction energies calculated between acetophenone and the rest of the system (receptor, lipids, water, ions) over time and the corresponding normalised energy distribution. b) Energies between acetophenone and the side chain of the K296 residue and the corresponding normalised energy distribution of the non-covalent energies. Panels c) and d) are analogous to a) and b), but were computed for pentanol. The pale coloured graphs show the original data whereas the darker lines represent averaged data over 50 time steps.

To learn more about the binding behaviour of the two odorants inside the receptor, their binding energies with the receptor, comprising electrostatic and van der Waals (vdW) components, were calculated over the entire simulation. Fig 4a and 4c feature the energies between the odorant molecules and the rest of the system, which includes the protein, the membrane and the surrounding water molecules and ions. The total binding energies of acetophenone and pentanol with the rest of the system are similar. This is supported by the normalized energy distribution for all non-covalent interactions shown in Fig 4. Interactions between the entire system and acetophenone are dominated by vdW interactions, while in the case of pentanol, electrostatic and vdW energies contribute almost equally. Fig 4b and 4d show the energies between the odorant molecules and the side chain of the amino acid K296. Apparently, the interactions between the side chain of K296 and the odorants are almost exclusively electrostatic as the vdW energies fluctuate around zero. Again, the flexible behaviour of pentanol inside the receptor should be noticed, as the energy distribution in Fig 4d behaves irregularly.

In summary, acetophenone shows stable long time binding inside the OR model studied here, while pentanol shows a much more flexible behaviour within the same receptor. In conclusion, in this specific case the chosen OR model is more suitable for binding acetophenone, and therefore, further calculations regarding the vibrational properties within the receptor were performed solely for acetophenone.

### Vibrational properties of acetophenone

**Fig 5:**
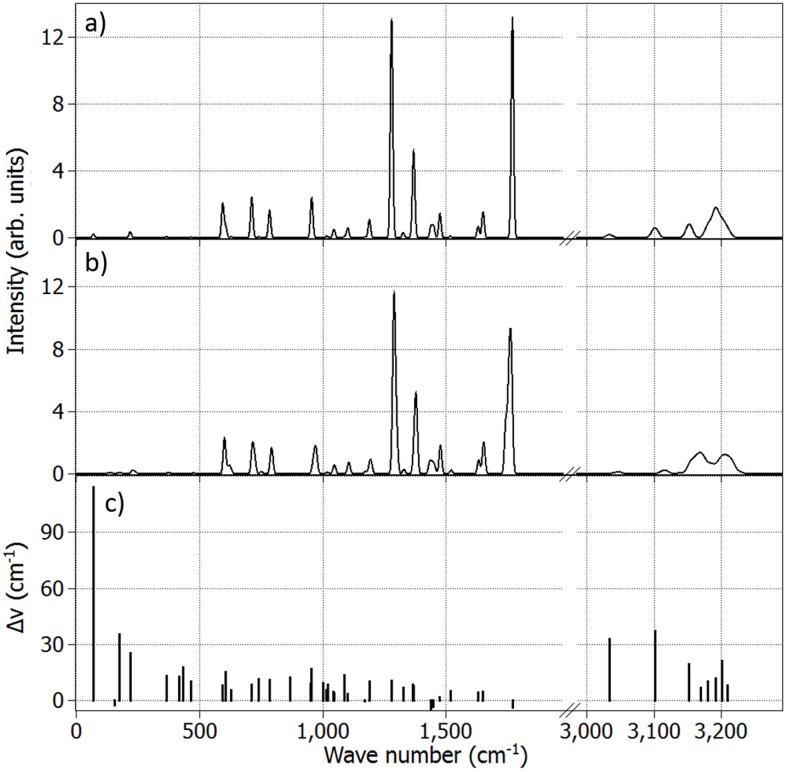
Infrared spectrum for acetophenone. a) in isolation and b) inside the protein computed as an average of 10 statistically independent MD configurations. Vibrational frequencies and intensities have been obtained at the B3LYP/cc-pVDZ level of theory. c) Energy shifts of different vibrational modes in acetophenone inside the receptor as compared to the vacuum case.

Fig 5 depicts the calculated infrared (IR) spectra of acetophenone in isolation (a), as well as inside the protein (b). The vibrational spectrum of the odorant is slightly modified by the receptor. Fig 5c shows the protein–vacuum shifts Δ*ν* (in cm^−1^) of the vibrational modes of acetophenone as a function of the in vacuum frequencies. These shifts are similar in magnitude to those found in an earlier study, comparing the influence of different solvents on the C = O stretch in acetophenone [33]. It should be noted that the change in vibrational frequency induced by the protein scaffold is of the order of 1 − 10 meV, which could not have any noticeable impact on the putative ET through the olfactory receptor and, therefore, is considered to be insignificant.

### Electron-phonon coupling between the receptor and the odorant

In the vibrationally-assisted mechanism, the olfactory event is thought to be triggered by an ET within the receptor. In that case, the receptor should be a system with at least two energetic states: one before the ET (a donor state with energy *E*_D_) and one after the ET (an acceptor state with energy *E*_A_). Due to the presence of the transferred electron either on the donor or the acceptor site of the OR the electric field is changed. Note, that there is still no ultimate evidence supporting or disputing this idea. In the present investigation we, therefore, assume a generic model of an electron donor/acceptor system that solely deals with the proof of principle.

The electron-phonon coupling between the two electronic states of the receptor and the odorant vibrations is quantified through the Huang-Rhys factor *σ*_*n*_, which, following the previously derived formalism [16, 30, 63], is given by

$$\sigma_{n}=\frac{{\left(u_{\text{D}}-u_{\text{A}}\right)}^{2n}}{n!\exp{\left(u_{\text{D}}-u_{\text{A}}\right)}^{2}}.$$ (1)

Here *n* is the vibrational state of the odorant molecule and *u*_*i*_ are the coupling strengths between the odorant vibration and the OR found in either the donor state (*i* = D) or the acceptor state (*i* = A). The coupling strengths *u*_*i*_ can be evaluated as

$$u_{i}\left(\mu \right)=-\sqrt{\frac{\omega_{\mu }}{2\hbar }}\cdot \delta \Pi_{\mu }\left(\vec{E_{i}}\right),$$ (2)

with the vibrational normal frequencies *ω*_*μ*_ and the mass weighted normal coordinate shift $\delta \Pi_{\mu }\left(\vec{E_{i}}\right)$ (in the units of $\sqrt{mass}\times length$), caused by the change in electric field $\vec{E_{i}}$ as due to the electron being located either on the donor or the acceptor site of the OR. The mass weighted normal coordinate shift $\delta \Pi_{\mu }\left(\vec{E_{i}}\right)$ can be calculated for both the donor and the acceptor states of the OR as

$$\delta \Pi_{\mu }\left(\vec{E_{i}}\right)=\sum_{n=1}^{N}\left(\sqrt{m(n)}\cdot y_{\mu }\left(n\right)\cdot \Delta \left(\vec{E_{i}},n\right)\right),$$ (3)

where summation is performed over all atoms of acetophenone, *m*(*n*) is the atomic mass, *y*_*μ*_ are the normal vibration coordinates of acetophenone in isolation and $\Delta (\vec{E_{i}},n)$ are the atomic displacements caused by the local electric field inside the receptor.

The Huang-Rhys factors *σ*_*n*_ can be readily calculated as soon as the vibrational spectrum {*ω*_*μ*_} and the normal vibrational coordinates for the odorant in isolation and inside the receptor before and after the ET are known, for example from *ab initio* calculations. A more complete derivation of the coupling strength and Huang-Rhys factor can be found in an earlier study [16].

In the present investigation the neutral receptor was considered to act as the donor state of the OR. The acceptor state was modelled by assigning radical character to amino acids, found at a reasonable distance of the odorant and having appropriate electron affinity potentials. The two amino acids Y268 and M86 shown in Fig 2 were chosen as suitable electron donor and acceptor candidates. While both being neutral before the ET (donor state), methionine was a radical anion and tyrosine a radical cation after the ET (acceptor state). We emphasize again that the selected pair of amino acids is used solely to determine the possible influence of ET dynamics on the bound odorant and to determine whether such a process could couple to odorant vibrations.

**Fig 6:**
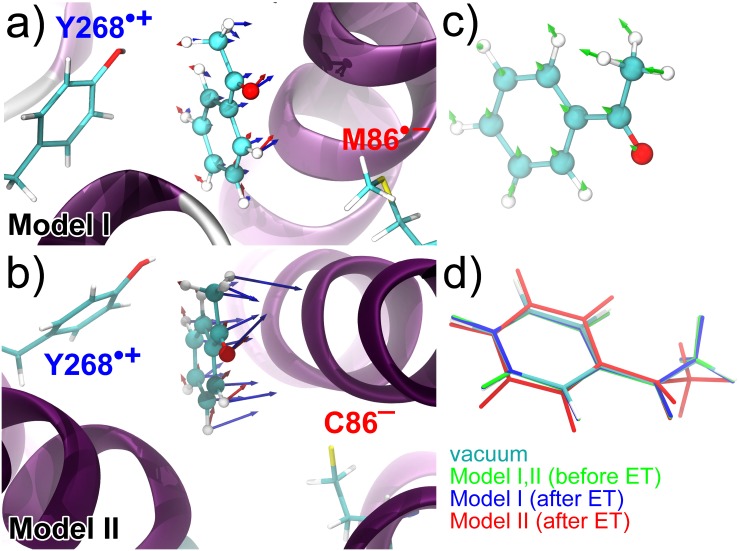
Electron transfer through the OR. Acetophenone at its binding site together with the two selected amino acid side chains acting as donor and acceptor residues. The putative donor-acceptor pair is featured through Model I and Model II corresponding to Y268^•+^/M86^•−^ a) and Y268^•+^/hC86^−^, respectively. b) Arrows indicate the electric field at the site of each atom calculated using the PE–DFT formalism. Red arrows are average electric fields over 10 uncorrelated MD configurations before the ET event, while blue arrows show those after ET for one MD configuration. c) The green arrows indicate the difference between the electric fields of the states before and after the ET in the case of Model I. d) Change of the equilibrium geometry of acetophenone upon ET in reference to the molecule in isolation (in vacuum). Green: before ET in the case of Model I and II; blue: after ET in the case of Model I; red: after ET in the case of Model II.

It is still largely unknown whether a suitable electron donor/acceptor pair exists in ORs, and the choice of a tyrosine/methionine pair is dictated by their rigid location in respect to acetophenone binding in the particular system studied here. Methionine does not possess the optimal electron affinity potential, and, therefore, could attract an additional electron depending on its environment: in unfavourable cases, methionine releases the ${\text{CH}}_{3}^{•}$ radical upon trapping an electron and turns into deprotonated homocysteine. We have thus considered two scenarios, for the electron donor/acceptor pair. ET model (I) involves the Y268^•+^/M86^•−^ pair and model (II) the Y268^•+^/hC86^−^ pair, as illustrated in Fig 6a and 6b.

**Fig 7:**
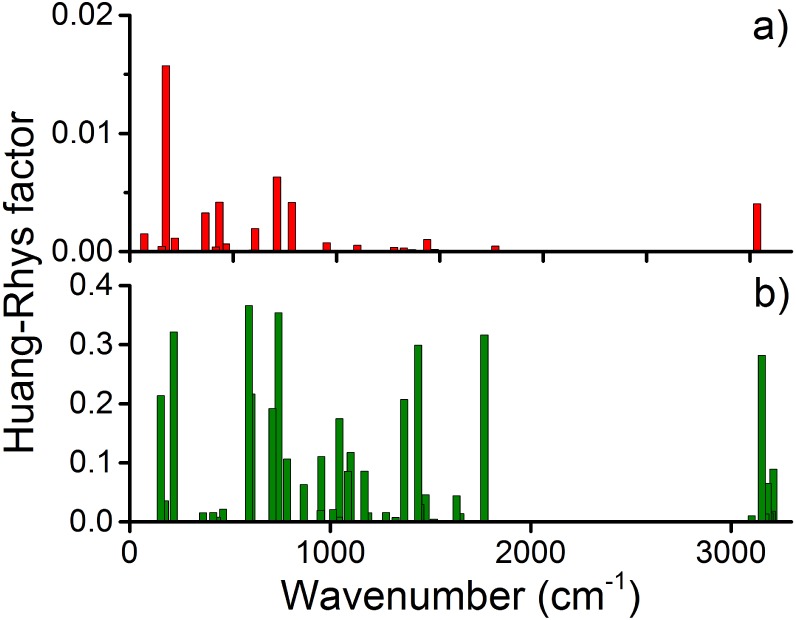
Electron-phonon couplings. Huang-Rhys factors as a function of the acetophenone vibrational frequencies calculated for a) Model I and b) Model II of the electron donor/acceptor pair, see Fig 6a and 6b.

**Fig 8:**
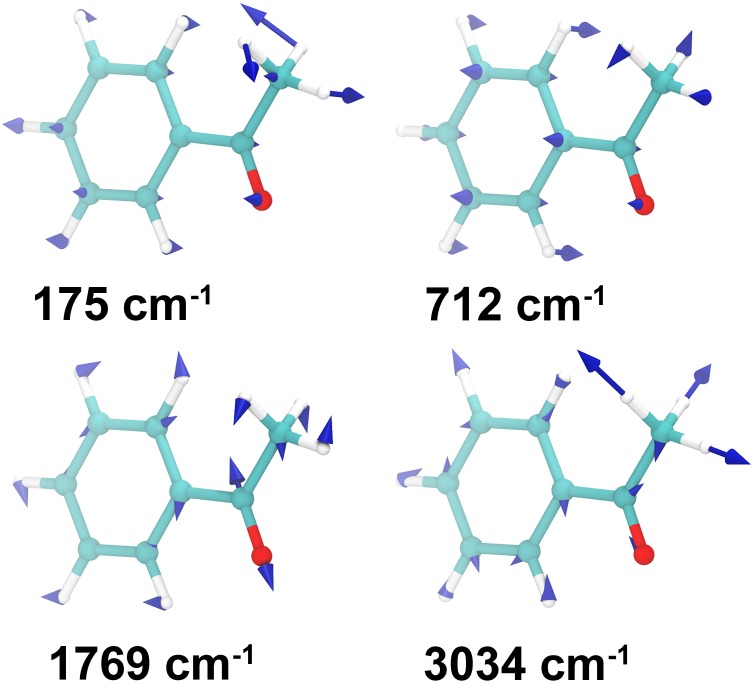
Acetophenone normal vibration modes. Visualization of several vibration modes in acetophenone which give the increased values for the Huang-Rhys factor inside the model receptor. The characteristic carbonyl stretch mode at 1769 cm^−1^ is also shown. The blue arrows and numbers correspond to the vibrational modes of the molecule in isolation.

The calculated values for the Huang-Rhys factors for the 45 vibrational modes of acetophenone in each model are given in Fig 7a and 7b. In Model I, the three vibrational modes of acetophenone which give the highest values are visualized in Fig 8 and correspond to the wavenumbers of 175 cm^−1^, 712 cm^−1^ and 3034 cm^−1^. Additionally, Fig 8 features the characteristic C = O stretch for acetophenone at 1769 cm^−1^ for which the Huang-Rhys factor is negligible in Model I, while being significant in Model II.

The comparison of Figs 5c and 7a does not reveal any significant correlation of the shifts in vibrational frequencies and the Huang-Rhys factor values. In Model I, the *ν* = 175 cm^−1^ mode is associated with *σ* = 0.016 and is by far the strongest electron-phonon coupling constant in acetophenone inside the protein in this case. This seems to be in good accordance with [16] where the strongest coupling for isolated acetophenone was also reported for a low-frequency mode. However, the Huang-Rhys factors for the high energy modes were about one order of magnitude higher than those found in the present investigation of Model I.

As follows from Fig 7b, The Huang-Rhys factors for Model II turn out to be significantly larger and exceed 0.3 for three vibrations. The actual values of the Huang-Rhys factors are thus strongly dependent on the given electron donor-acceptor pair, and the precise values of the Huang-Rhys coupling coefficient can only be established if such a pair is realized in the real receptor. The large difference in the Huang-Rhys factor values arising between Model I and Model II is due to the increase of the electrostatic potential in the acetophenone binding cavity upon releasing the ${\text{CH}}_{3}^{•}$ radical from M86. The increased electrostatic potential leads to significant perturbations of the equilibrium geometry of acetophenone upon ET in reference to the molecule in isolation as demonstrated in Fig 6d. Note that the change in the equilibrium geometry is minimal in Model I, resulting in the fairly small values of the Huang-Rhys factors in this case.

It is also important to stress that the presently observed deviations of the Huang-Rhys factors in Model I to previous calculations [16] arise due to the simplicity of the earlier model calculations. In particular, the difference in the electric field generated upon the ET event, which in Model I turns out to be smaller than the value estimated earlier [4, 16]. The local electric fields acting on acetophenone before and after the ET in Model I are shown in Fig 6a. The average electric field at the center of acetophenone is 0.0102 au before the ET event and 0.0116 au after. In the previous studies [4, 16] the induced field of a receptor was estimated to be 0.01 au, while the ET was simulated by having an electric field of 0.01 au before and −0.01 au after the ET applied to acetophenone along its dipole moment, leading to a field change of 0.02 au. Fig 6c displays the difference in electric fields for acetophenone inside the model receptor between the two ET states in Model I. The magnitude of this difference is 0.0087 au, being directed somewhat out of the plane of the molecule, i.e. it is not collinear with the dipole moment of acetophenone. The presently calculated average electric field difference for acetophenone inside the receptor is about three times smaller than the one estimated earlier [16], which partly explains the smaller Huang-Rhys factors.

The performed analysis reveals that the electric field arising upon the ET should not be assumed to be directed along the dipole moment of the odorant, as it would be severely constrained. Once the electric field difference increases, as for example happens in Model II where it becomes 0.028 au, the misalignment of the molecular dipole moment with the local electric field results in significant geometrical perturbations of the equilibrium structure of acetophenone, which is finally manifested through the increased values of Huang-Rhys factors for some vibrational modes.

### Dynamical behaviour of acetophenone after the ET

**Fig 9:**
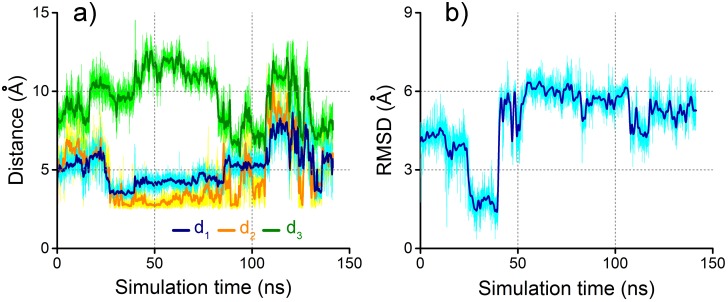
Acetophenone unbinds after the ET event. Time evolution of the distances *d*_1_, *d*_2_ and *d*_3_ between acetophenone and side chains of amino acids K296, Y268 and M86 inside the receptor, as defined in Fig 2a. a) The dynamics of distances is recorded immediately after the putative ET event as modeled in Model II represented through the Y268^•+^/hC86^−^ donor/acceptor pair, see Fig 6b. b) Time evolution of the RMSD calculated for acetophenone after ET event in Model II.

To illustrate the possible impact of an ET reaction in the OR we have employed MD simulations to study the dynamics of acetophenone in the binding pocket after the ET event, as proposed in Model II, see Fig 6b. Fig 9a illustrates the time evolution of distances between acetophenone and some of amino acid residues that coordinate its binding before the ET, Fig 9b shows the time evolution of acetophenone RMSD, as calculated relatively to the position prior ET. The figure shows that the binding of acetophenone inside the receptor is no longer stable as the three distances behave highly irregularly and the RMSD increases substantially. It is interesting that the unstable behavior of acetophenone manifests itself instantaneously after the ET event, indicating an eventual departure of the odorant.

An important quantity of an ET event is the receptor reorganisation energy. This quantity is especially crucial for the vibrationally-assisted mechanism of olfaction, as a large value of the reorganisation energy would render this mechanism physically impossible [16, 18]. Below we estimate the reorganisation energy for the studied OR model by analysing the results of MD simulations before and after the ET reaction. Note, that this analysis serves solely as an order of magnitude estimate of the reorganisation energy. More accurate calculations [64–66] would only be possible and conclusive once an actual structure of an OR is revealed.

The reorganisation energy follows from the Marcus theory of electron transfer [64, 67], which predicts the ET rate constant as

$$k=\frac{2\pi }{\hbar }{\left|H_{DA}\right|}^{2}\frac{1}{\sqrt{4\pi \lambda k_{B}T}}\text{exp}\left(-\frac{{\left(\lambda +\Delta G\right)}^{2}}{4\pi \lambda k_{B}T}\right).$$ (4)

Here *H*_*DA*_ is the electronic coupling between the donor and acceptor states participating in the electron transfer reaction, *λ* is the reorganization energy of the receptor and Δ*G* is the driving force of the electron transfer reaction. *T* is the temperature, *k*_*B*_ is the Boltzmann constant, and ℏ is the Planck constant.

The reorganisation energy *λ* could be established through free energy perturbation calculations [68, 69], however, these calculations would not be sufficiently conclusive without an accurate atomistic structure of the receptor. Alternatively, one could estimate the order of magnitude of *λ* by sampling the potential energy of the OR before and after the ET event. The potential energy of the receptor follows the gaussian probability density distribution, which reads as

$$p\left(E_{i}\right)=\frac{1}{\omega_{i}\sqrt{\pi /2}}\exp\left(-\frac{2{\left(E_{i}-\left\langle E_{i}\right\rangle \right)}^{2}}{\omega_{i}^{2}}\right),$$ (5)

where the subscript *i* indicates the state of the receptor (before or after the ET event), *E* denotes the potential energy of the OR, 〈*E*〉 is the average potential energy value, and *ω* is the width of the probability density distribution.

**Fig 10:**
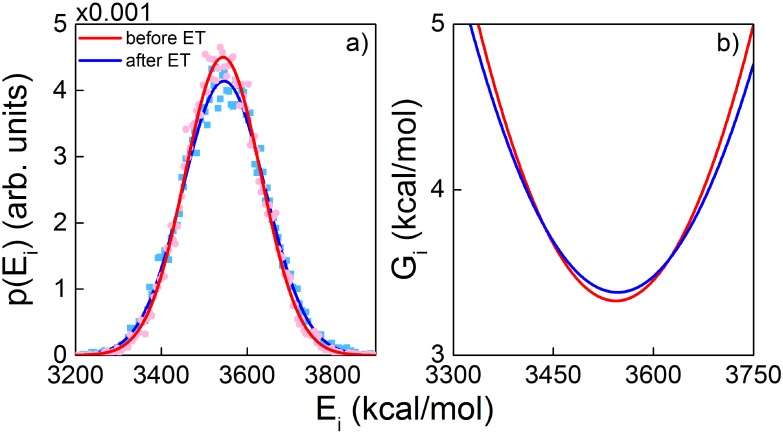
Reorganization energy of the OR model. a) The probability density distribution of the OR potential energy before (red) and after (blue) the ET event. The after ET event corresponds to the data obtained for Model II, see Fig 6b. Points indicate data obtained from MD simulations, while lines correspond to the normalized gaussian fitting function introduced in Eq (5). b) Free energy profiles for OR before (red) and after (blue) ET, computed using Eq (6) from the potential energy probability density distribution.

The potential energy probability density functions for the OR before and after the ET event are shown in Fig 10a by red and blue lines respectively. These distributions follow directly from the performed MD simulations, where the potential energy of the entire receptor was sampled and analysed. The width of the sampling bins was set to 5 kcal/mol, and the gaussian-shaped function Eq (5) was then fitted to the resulting data points. The 150 ns long MD trajectories turn out to be long enough to provide sufficient statistics to have a smooth energy distribution profile in both redox states of the OR. It is striking that for both states the distributions turn out to be very close to each other, indicating that an ET event has a minor impact on the potential energy of the OR model.

To estimate the reorganisation energy from the energy distribution functions, one should calculate the free energy of the OR in a given redox state as

$$G_{i}=-RT\ln\left\{p\left(E_{i}\right)\right\},$$ (6)

where *R* = 0.001987 kcal/(mol⋅K) is the universal gas constant, *T* = 310 K, is the temperature used for the estimates.

Fig 10b shows the calculated free energies for the studied OR calculated as a function of the OR potential energy. The two parabolic profiles are almost indistinguishable having their minima at a low value of *G*, which suggests that for the studied OR model the reorganisation energy is very small, i.e. ≪ 1 kcal/mol. This conclusion is highly important for the vibrationally assisted mechanism of olfaction, as it shows that for a model receptor low reorganisation energies are, in principle, possible. The result should, however, not be generalized for all OR yet.

## Discussion and Outlook

To get insight into the controversially discussed mechanism of olfaction, we considered the suggested theory based on a vibrationally-assisted ET being a trigger for the primary olfactory event [15], and studied the influence of a putative OR on the vibrational properties of an odorant inside this receptor. Presently no crystal structures of ORs are available which makes all theoretical studies of the olfactory mechanism rather model dependent. A promising homology model for ORs based on the rhodopsin apoprotein was recently suggested [29] and is employed in the present investigation.

First we studied the binding specificity of the OR model toward pentanol and acetophenone as odorant molecules through MD simulations and demonstrated long time binding of acetophenone. Subsequently the effects of the OR on the vibrational properties of the odorant acetophenone were studied. IR spectra of isolated acetophenone and acetophenone inside the receptor were computed and compared. The changes in vibrational frequencies induced by the protein turned out to be of the order of 1 − 10 meV, which is expected to have no significant impact on the putative ET in the receptor.

Furthermore, Huang-Rhys factors were calculated for each vibrational mode of acetophenone. There seems to be no noticeable correlation between the mode’s frequency shift and the computed values for the Huang-Rhys factor. The calculated Huang-Rhys factors are highly dependent on the actual electron donor/acceptor pair inside the receptor. We showed that the suggested values for the Huang-Rhys factors in earlier estimates [4] can be reconciled with an all-atom structure of the receptor, being, however, too small to be detectable [18]. Alternation of the electron acceptor could lead to a more than order of magnitude increase of the Huang-Rhys factor resulting from an increased electrostatic field in the binding pocket of the receptor.

The specific choice of a Y268/M86 pair of amino acid residues to serve as electron donor and acceptor was dictated by the necessity of these putative redox partners to embrace the bound odorant molecule. It should be noted that if ORs are powered by ET events, the acceptor residue of the primary electron transfer reaction could in fact be some sort of a relay residue, which temporarily hosts an electron before transferring it further to some redox centre of the receptor, as proposed originally [15]. The choice of a methionine residue is especially interesting in this respect, as this amino acid has been suggested to be involved in relay electron transfers [70]. Such a multistep ET and possible followup events are interesting to investigate in the future, once a better OR structure would become available.

In this investigation, we have addressed some of the unanswered questions in the current state of theoretical descriptions of vibrationally-assisted ET in ORs, recently pointed out by Block *et al.*[18]. The bonding characteristics between odorant and the model OR are no longer neglected and were studied here in detail. Furthermore, dynamical fluctuations were considered and are shown to have no impact on the the average IR spectrum in the case of acetophenone, being one of the prominent odorants studied [6, 7, 16, 17].

A particularly important parameter for the viability of the vibrationally-assisted theory of olfaction is the receptor reorganization energy, which we have estimated here. It turned out that an ET in a membrane bound receptor could occur with a negligible reorganisation energy. Although highly surprising, we consider the latter result as an important indication that low reorganisation energies in biological systems are possible. Nevertheless, due to the model-nature of the studied OR model this conclusion is too premature to be applied to real OR, and should be reevaluated once the atomistic structures of ORs would be revealed.

We showed that a receptor influences the vibrational properties of an odorant even though the impact is seemingly negligible. On contrary, an ET event could significantly affect the equilibrium geometry of the bound odorant and its binding stability. This conclusion, however, remains largely speculative: At present, it is unknown whether any olfactory receptor has a pair of amino acid residues that could facilitate an ET reaction, as no OR has been crystallised yet. We believe that our investigation illuminates the physical viability of the vibrationally-assisted mechanism of olfaction and will stimulate further endeavours for revealing structures of these important receptors, at least in some organisms.

## Supporting Information

S1 File CHARMM topology file for positively charged tyrosine residue. (PDF)

S2 File CHARMM parameter file for positively charged tyrosine residue. (PDF)

S3 File CHARMM topology file for negatively charged cysteine residue. (PDF)

S4 File CHARMM parameter file for negatively charged cysteine residue. (PDF)
