# Supplementary material for: How Far Does a Receptor Influence Vibrational Properties of an Odorant?
Source: PLoS One. 2016 Mar 25;11(3):e0152345. doi: 10.1371/journal.pone.0152345 (PMC4807836; doi:10.1371/journal.pone.0152345)
Supplement: S1 File — (PDF) [file pone.0152345.s001.pdf]

```
MASS     1 CT2P   12.01100  C
MASS     1 HA2P    1.00794  H
MASS     1 CAP    12.01100  C
MASS     1 HPP     1.00794  H
MASS     1 OH1P   15.99900  O
MASS     1 HP      1.00794  H

AUTO ANGLES DIHE

RESI TYRP         1.00
GROUP
ATOM N    NH1    -0.47        !     |        HD1  HE1
ATOM HN   H       0.31        !  HN-N         |    |
ATOM CA   CT1     0.07        !     |   HB1  CD1--CE1
ATOM HA   HB1     0.09        !     |   |   //      \\
GROUP                         !  HA-CA--CB--CG      CZ--OH
ATOM   CB CT2P   -0.05        !     |   |    \  __  /     \
ATOM  HB1 HA2P    0.09        !     |   HB2  CD2--CE2     HH
ATOM  HB2 HA2P    0.09        !   O=C         |    |
ATOM   CG  CAP    0.13        !     |        HD2  HE2
ATOM  CD1  CAP   -0.06
ATOM  HD1  HPP    0.25
ATOM  CE1  CAP   -0.36
ATOM  HE1  HPP    0.19
ATOM   CZ  CAP    0.74
ATOM   OH OH1P   -0.79
ATOM   HH   HP    0.75
ATOM  CD2  CAP   -0.06
ATOM  HD2  HPP    0.25
ATOM  CE2  CAP   -0.36
ATOM  HE2  HPP    0.19
GROUP
ATOM C    C       0.51
ATOM O    O      -0.51
BOND CB HB1  CB HB2  CB CG
BOND CG CD2  CG CD1  CD1 CE1
BOND CD1 HD1  CE1 CZ  CE1 HE1  CZ CE2
BOND CZ OH  OH HH  CD2 CE2  CD2 HD2
BOND CE2 HE2
BOND CB  CA
BOND N   HN   N   CA    C   CA   C   +N
DOUBLE   O   C
IMPR N -C CA HN  C CA +N O
CMAP -C  N  CA  C   N  CA  C  +N

END
```
