# Supplementary material for: How Far Does a Receptor Influence Vibrational Properties of an Odorant?
Source: PLoS One. 2016 Mar 25;11(3):e0152345. doi: 10.1371/journal.pone.0152345 (PMC4807836; doi:10.1371/journal.pone.0152345)
Supplement: S2 File — (PDF) [file pone.0152345.s002.pdf]

```
BONDS
!V(bond) = Kb(b - b0)**2
!
!Kb: kcal/mole/A**2
!b0: A
!
!atom type Kb b0
!
CT2P  HA2P  344.710    1.108
CT2P  CAP   237.868    1.487
CAP   CAP   388.999    1.367
CAP   HPP   366.214    1.077
CAP   OH1P  386.455    1.386
OH1P  HP    516.619    0.958
!backbone
CT2P  CT1   222.500    1.538

ANGLES
!
!V(angle) = Ktheta(Theta - Theta0)**2
!
!V(Urey-Bradley) = Kub(S - S0)**2
!
!Ktheta: kcal/mole/rad**2
!Theta0: degrees
!Kub: kcal/mole/A**2 (Urey-Bradley)
!S0: A
!
!atom types     Ktheta    Theta0   Kub     S0
!
CT2P  CAP   CAP   61.531   125.304
HA2P  CT2P  HA2P  58.720   112.067
HA2P  CT2P  CAP   50.923   115.481
CAP   CAP   HPP   46.906   120.661
CAP   CAP   CAP   122.230  120.041
CAP   CAP   OH1P  93.872   125.447
CAP   OH1P  HP    104.007  112.774
!backbone
NH1   CT1   CT2P  70.000   113.500
HA2P  CT2P  CT1   26.500   110.100   22.53   2.17900
CT1   CT2P  CAP   51.800   107.5000
HB1   CT1   CT2P  35.000   111.000
CT2P  CT1   C     52.000   108.000


DIHEDRALS
!
!V(dihedral) = Kchi(1 + cos(n(chi) - delta))
```

```
!Kchi: kcal/mole
!n: multiplicity
!delta: degrees
!
!atom types             Kchi    n   delta
!
HPP   CAP   CAP   OH1P  2.5220   2  180.00
HPP   CAP   CAP   HPP   0.0870   2  180.00
HA2P  CT2P  CAP   CAP   0.0180   6  0.00
CAP   CAP   OH1P  HP    1.0380   2  180.00
CAP   CAP   CAP   OH1P  0.0000   2  180.00
CAP   CAP   CAP   HPP   1.3500   2  180.00
CAP   CAP   CAP   CAP   1.6500   2  180.00
CT2P  CAP   CAP   HPP   1.0130   2  180.00
CT2P  CAP   CAP   CAP   1.5570   2  180.00
!backbone
H     NH1   CT1   CT2P  0.0000  1     0.00
CAP   CT2P  CT1   NH1   0.5200  1   180.00
CAP   CT2P  CT1   NH1   0.6200  2   180.00
CAP   CT2P  CT1   NH1   1.5800  3     0.00
NH1   CT1   CT2P  HA2P  0.2000  3     0.00 ! From X    CT1  CT2  X
HB1   CT1   CT2P  CAP   0.2000  3     0.00 ! From X    CT1  CT2  X
HB1   CT1   CT2P  HA2P  0.2000  3     0.00 ! From X    CT1  CT2  X
O     C     CT1   CT2P  1.4000  1     0.00
CT2P  CT1   NH1   C     1.8000  1     0.00 ! ALLOW PEP
NH1   C     CT1   CT2P  0.0000  1     0.00 !   ALLOW PEP
X     CT1   CT2P  X     0.2000  3     0.00 ! ALLOW   ALI
X     CT2P  CAP   X     0.0000  6     0.00 ! ALLOW   ALI ARO


NONBONDED nbxmod  5 atom cdiel shift vatom vdistance vswitch -
cutnb 14.0 ctofnb 12.0 ctonnb 10.0 eps 1.0 e14fac 1.0 wmin 1.5
!
!V(Lennard-Jones) = Eps,i,j[(Rmin,i,j/ri,j)**12 -
2(Rmin,i,j/ri,j)**6]
!
!epsilon: kcal/mole, Eps,i,j = sqrt(eps,i * eps,j)
!Rmin/2: A, Rmin,i,j = Rmin/2,i + Rmin/2,j
!
!atom  ignored    epsilon      Rmin/2   ignored   eps,1-4
Rmin/2,1-4
!
CAP   0.0  -0.070000  1.992400
CT2P  0.0  -0.056000  2.010000  0.0  -0.010000  1.900000
HA2P  0.0  -0.034000  1.340000
HPP   0.0  -0.030000  1.358200  0.0  -0.030000  1.358200
OH1P  0.0  -0.152100  1.770000

END
```
