# Supplementary material for: How Far Does a Receptor Influence Vibrational Properties of an Odorant?
Source: PLoS One. 2016 Mar 25;11(3):e0152345. doi: 10.1371/journal.pone.0152345 (PMC4807836; doi:10.1371/journal.pone.0152345)
Supplement: S4 File — (PDF) [file pone.0152345.s004.pdf]

```
BONDS
!V(bond) = Kb(b - b0)**2
!
!Kb: kcal/mole/A**2
!b0: A
!
!atom type Kb b0
!
CT2N  CT2N  299.988    1.537
CT2N  HA2N  340.237    1.098
CT2N  SN    208.184    1.834
!backbone
CT2N  CT1   222.500    1.538


ANGLES
!
!V(angle) = Ktheta(Theta - Theta0)**2
!
!V(Urey-Bradley) = Kub(S - S0)**2
!
!Ktheta: kcal/mole/rad**2
!Theta0: degrees
!Kub: kcal/mole/A**2 (Urey-Bradley)
!S0: A
!
!atom types     Ktheta    Theta0   Kub     S0
!
!
CT2N  CT2N  HA2N  49.490   108.195
CT2N  CT2N  SN    64.789   115.974
HA2N  CT2N  HA2N  51.870   110.134
HA2N  CT2N  SN    40.644   111.988
!backbone
NH1   CT1   CT2N  70.000   113.500
HA2N  CT2N  CT1   26.500   110.100   22.53   2.17900
CT2N  CT2N  CT1   58.350   113.500   11.16   2.56100
HB1   CT1   CT2N  35.000   111.000
CT2N  CT1   C     52.000   108.000


DIHEDRALS
!
!V(dihedral) = Kchi(1 + cos(n(chi) - delta))
!
!Kchi: kcal/mole
!n: multiplicity
!delta: degrees
!
```

```
!atom types              Kchi    n   delta
!
HA2N  CT2N  CT2N  SN    0.3750   3  0.00
HA2N  CT2N  CT2N  HA2N  0.1590   3  0.00
!backbone
H     NH1   CT1   CT2N  0.0000  1     0.00
CT2N  CT2N  CT1   NH1   0.8800  1   180.00
NH1   CT1   CT2N  HA2N  0.2000  3     0.00 ! From X    CT1  CT2  X
HB1   CT1   CT2N  CT2N  0.2000  3     0.00 ! From X    CT1  CT2  X
HB1   CT1   CT2N  HA2N  0.2000  3     0.00 ! From X    CT1  CT2  X
O     C     CT1   CT2N  1.4000  1     0.00
CT2N  CT1   NH1   C     1.8000  1     0.00 ! ALLOW PEP
NH1   C     CT1   CT2N  0.0000  1     0.00 !   ALLOW PEP
X     CT1   CT2N  X     0.2000  3     0.00 ! ALLOW   ALI
X     CT2N  CT2N  X     0.1900  3     0.00



NONBONDED nbxmod  5 atom cdiel shift vatom vdistance vswitch -
cutnb 14.0 ctofnb 12.0 ctonnb 10.0 eps 1.0 e14fac 1.0 wmin 1.5
!
!V(Lennard-Jones) = Eps,i,j[(Rmin,i,j/ri,j)**12 -
2(Rmin,i,j/ri,j)**6]
!
!epsilon: kcal/mole, Eps,i,j = sqrt(eps,i * eps,j)
!Rmin/2: A, Rmin,i,j = Rmin/2,i + Rmin/2,j
!
!atom  ignored    epsilon      Rmin/2   ignored   eps,1-4
Rmin/2,1-4
!
CT2N  0.0  -0.056000  2.010000  0.0  -0.010000  1.900000
HA2N  0.0  -0.034000  1.340000
SN    0.0  -0.450000  2.000000

END
```
