# Supplementary material for: How Far Does a Receptor Influence Vibrational Properties of an Odorant?
Source: PLoS One. 2016 Mar 25;11(3):e0152345. doi: 10.1371/journal.pone.0152345 (PMC4807836; doi:10.1371/journal.pone.0152345)
Supplement: S3 File — (PDF) [file pone.0152345.s003.pdf]

```
MASS     1 CT3N  12.01070  C
MASS     1 HA3N   1.00794  H
MASS     1 CT2N  12.01070  C
MASS     1 HA2N   1.00794  H
MASS     1 SN    32.06500  S

AUTO ANGLES DIHE

RESI METN    -1.0         !     |
GROUP                     !  HN-N
ATOM N    NH1    -0.47    !     |   HB1 HG1
ATOM HN   H       0.31    !     |   |   |
ATOM CA   CT1     0.07    !  HA-CA--CB--CG--SD
ATOM HA   HB1     0.09    !     |   |   |
GROUP                     !     |   HB2 HG2
ATOM C    C       0.51    !   O=C
ATOM O    O      -0.51    !     |
GROUP
ATOM   CB CT2N   -0.29
ATOM  HB1 HA2N    0.09
ATOM  HB2 HA2N    0.09
ATOM   CG CT2N   -0.26
ATOM  HG1 HA2N    0.09
ATOM  HG2 HA2N    0.09
ATOM   SD   SN   -0.81
BOND CB CG  CB HB1  CB HB2
BOND CG HG1  CG SD  CG HG2
BOND CB CA
BOND N  HN   N  CA    C  CA   C  +N
DOUBLE   O  C
IMPR N -C CA HN  C CA +N O
CMAP -C  N  CA  C   N  CA  C  +N
END
```
